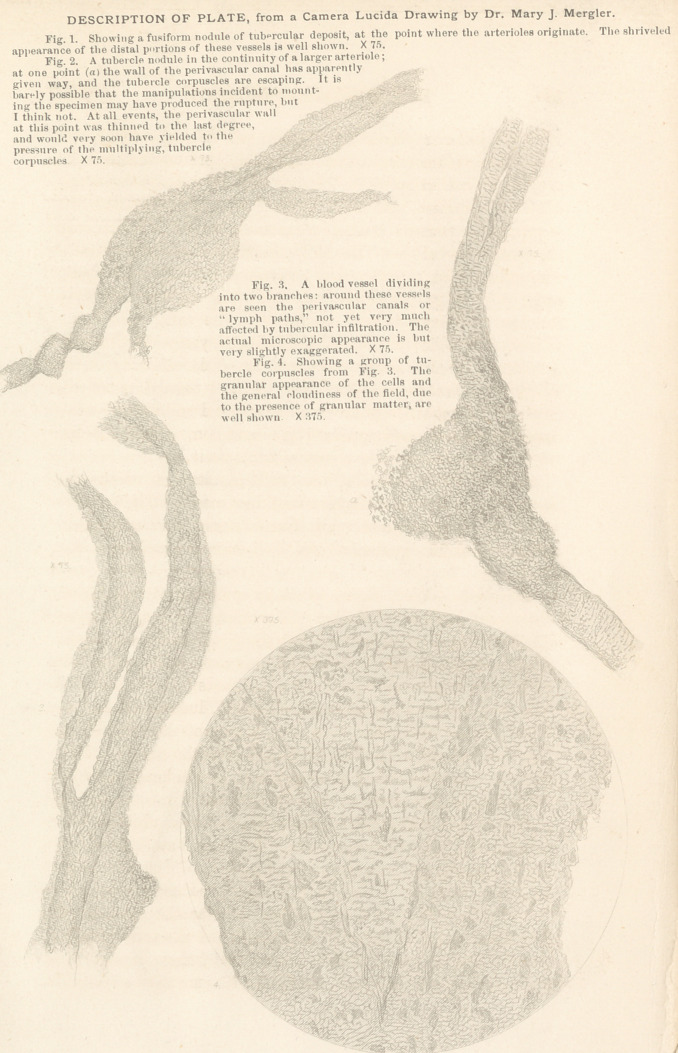# Tubercular Meningitis

**Published:** 1879-07

**Authors:** Chas. W. Earle

**Affiliations:** Prof. of Diseases of Children, Woman’s Medical College, Chicago


### ARTICLE II.

TUBERCULAR MENINGITIS. By CHAS. W. EARLE, M.D., Prof. of Diseases of Children, Woman's Medical College, Chicago.

War, pestilence and famine have been regarded as the three most dreadful calamities which may befall a race.

If a student of those causes which consign more than one-fifth of all born into the world to early graves before the end of the first year, and one in three before the completion of the fifth year, were asked to enumerate the three principal classes of infantile diseases contributing in the greatest degree to this terrible destruction of life, he would in all probability say:

1st. Diseases of the throat and larynx, in which stenosis is the principal symptom.

2d. Entero-colitis with its consequent sequelæ of mal-nutrition; and

3d. Cerebral diseases, among which is found the quite frequent and almost uniformly fatal malady termed tubercular meningitis.

To this disease, and more particularly to the consideration of its causes, pathology, diagnosis and prophylactic treatment, I have the honor to call your attention this evening.

Much confusion has been caused by the different synonyms of this disease, and confounding with it simple meningitis. The differentiation between simple and tubercular meningitis I shall notice and attempt to establish, as far as possible, at another place in this paper. The different terms which have been used to convey the idea we now mean by tubercular meningitis, I will notice and dispose of at this time:

Acute hydrocephalus, basilar meningitis, hydrops cerebri, carus hydrocephalus, Whytt's disease, water on the brain, water brain fever.

This list could easily be enlarged, but the variety of terms already given is sufficient to show the great diversity of opinion which has existed in regard to a disease which at the present day is quite generally conceded to be due to abnormal cell proliferation, and may take place not only in the subject having a tubercular taint, but in a perfectly healthy child, who has been subjected to any of those causes which will produce rapid and imperfect or atypical cell growth.

The symptoms of this disease have been divided into three stages. Such a division must necessarily suffer change, but in the study of the disease, it is perhaps well to recognize them.

First, is the stage of *irritation.* The symptoms present at this time are variable and peculiar, and will in all probability only be recognized in those having distinct symptoms of tuberculosis, usually of the pulmonary variety. There is progressive emaciation, particularly marked in the body and wanting in the face, sometimes headache but more frequently vertigo and unsteadiness in walking, and in many cases constipation, or alternate diarrhœa and constipation, usually however preceding any symptoms referable to the head we find a slight fever in the afternoon, and in most of the cases vomiting. The fever is decidedly remittent, and the vomiting, unless seen by the physician, is easily explained by the attendants as caused by some slight indigestion, or due to biliousness, a favorite disease, as you all know, among the people. The fever is very liable to mislead, and taken without a careful consideration of all other symptoms, the disease at this time may be diagnosticated as infantile remittent. I have made this mistake myself, and have seen it occur in the practice of those older and possessing far greater diagnostic powers than I do. The vomiting is cerebral usually from the first; it is forcible, ejected to some distance from the mouth, not, as in indigestion, attended with nausea, eructations and the like, but expelled with great force with none of those concomitant symptoms. During the close of this stage, which in my experience lasts from 6 to 10 days, the child becomes feverish and very irritable. It throws itself about from place to place, is contented only for a moment in its mother's arms, and then only for a moment in the arms of the next attendant. It throws its head and arms wildly about, and the good natured and happy little one becomes cross and exceedingly restless. In many cases, especially those who have been relatively healthy and not the subjects of the tubercular or scrofulous diathesis, and yet who die of tubercular meningitis developed in a manner and from causes I shall presently enumerate, these symptoms of progressive emaciation are not present. The first noticeable symptom, in many cases, will be a periodical fever, irritability and vomiting.

The second stage is that of *pressure.* The pupils, even before any other sign of this stage is present, will, if noticed carefully, be found contracted, but after the symptoms of this stage are fairly inaugurated, they will usually be found to be dilated. The child has become more quiet; there is in the early part of this stage occasionally a vacant stare, which in the course of a few days is succeeded by fixedly dilated pupils, which fail to respond to light, and in many cases absolute want of sight. The child may now be partially comatose at times, and convulsions and contractions often take place. Indeed, convulsions, either partial or complete, are liable to occur throughout the disease; also contortions of the muscles of the face, and grinding of the teeth. In one or two cases I have noticed a tendency to contraction of the muscles of the neck, and indeed this was the particular symptom in one case which alarmed the mother, after the restlessness and irritability of a week, which had been thought to be incident to teething, was quieted, and the mother was laboring under the delusion that her little one was improving. The quiet was coma, and the child was in the midst of tubercular meningitis. The pulse, which may have been somewhat slow, has at this time usually increased in frequency, and toward the commencement of the third stage begins to be irregular. This symptom has in a few cases been the first of brain pressure.

The third stage is that of *paralysis.* Coma increases — the pulse is very rapid and markedly irregular — the respirations are sighing — there is difficulty, if not inability, to swallow — the surface of the body, while hyperæsthetic at first, now comes to be anæsthetic — prostration increases — the eyes are covered with a film, and death closes the scene.

During all this I never have discovered anything particularly useful from the temperature or from observation with the ophthalmoscope. The value of the last named instrument, however, in the hands of other observers, I will notice at another place in this paper. In the recapitulation of symptoms given above, I have necessarily omitted many, and have confined myself to the picture of the disease pursuing a normal course.

Following I append a list of eleven cases, from which I deduce the results and opinions given in this paper.

I call particular attention to the family history, and to the indications of *trauma* as a cause of the disease.

*Cases.*

| *Name.* | *Age.* | | *Duration.* | |
|---|---|---|---|---|
| *Gracie N. | 18 mos. | Scrofulous diathesis or taint. | 2 days. | Father very healthy. Mother had enlarged glands at birth of this child, but since has become much stronger, and two children since born are apparently perfectly healthy. |
| Ida N. | 11 mos. | Tubercular diathesis. | 14 days. | Father died of consumption. |
| Nellie G. | 3 yrs. | Scrofulous diathesis otitis. | 15 days. | Both parents young and small. Purulent discharge from ears for months. |
| Walter G. | 30 mos. | Scrofulous diathesis otitis. | 11 days. | Otitis existed in father when young — a kind of chronic family complaint. |
| Charley V. V. With Dr. Jones. | 15 mos. | Tubercular diathesis. | 14 days. | Father in all probability has phthisis — so stated by family physician. |
| Maud C. | 2 yrs. | Fell down stairs. | 20 days. | No taint apparent. Father and mother were healthy. The child never had any prostrating disease. |
| Rollie P. | 1 yr. 6 m. | Scrofulous diathesis | 5 days. | Scrofula 2nd generation back. |
| Annie L. A. | 2 yrs. | Fell down stairs. | 1 month. | No taint apparent. Father and mother both dark complexion, Germans, and perfectly healthy. |
| Lilian N. With Prof. Danforth. | 2 yrs. 9 m. | Fell down stairs. | 16 days. | No taint apparent. |
| Thomas C. | 15 yrs. | Necrosis tibia. | Symptoms referable to brain 10 days. | |

* It is quite probable that this case was acute non-tubercular meningitis.

*Cases—Continued.*

| *Name.* | *Age.* | | *Duration.* | |
|---|---|---|---|---|
| Emma G. | 9 mos. | Tubercular diathesis. | 4 weeks. | Mother had three sisters die from water on brain. Commenced to emaciate 4 weeks before death; constipation; cross and irritable; fever p. m.; *cerebral tache;* head reracted toward side. Pulse 135, temperature 101, 5 days before death. Pulse 190, 2 days before death. |

In the majority of these cases we have evidence of the tubercular or scrofulous diathesis, and from the usual and accepted ideas of pathology we should have no trouble in divining the cause of the tubercular meningitis taking place in children born of parents with this taint.

The following case, No. 9 in the list, I give in full for several reasons. It was in a healthy family; there was an injury; a *post mortem* was permitted and tubercle was found. It illustrates one of a few cases which have come under my observation where, with our previously accepted ideas of pathology, it is difficult to trace the cause of the disease. I have discovered no new pathology; but we have here a field for study.

This case, then, I give in full, particularly the result of the *post mortem.* It occurred in the practice of Prof. I. N. Danforth, and was published by him in the *American Quarterly Microscopical Journal.* I saw the case in consultation, and was present at the autopsy.

Lilian N. came under Dr. Danforth's care April 4th, 1878. She was two years and nine months old, and had been, up to a short time previous, a bright, active and well-developed child, with a good degree of strength and health. She had blue eyes, a light complexion, and a fair, transparent skin. Some three or four months previous she fell downstairs, and received a slight contusion over the right parietal bone. It was not regarded serious, however, and was soon forgotten.

A few weeks later, members of the family began to observe that the child manifested unusual irritability and fretfulness, this being more noticeable from the fact that the little one had always been a remarkably cheerful and happy child. Her wonted cheerfulness and vivacity, however, were now replaced by depression and irritability. She would cry long and vigorously upon the smallest and most absurd provocation, and she was acquiring an unusual and unaccountable temper. At this time the doctor was called. The child presented these peculiarities of disposition: had a fever every afternoon, and had the appearance of "being moderately sick and very nervous and cross from an ordinary infantile remittent." The case rapidly grew worse, and the doctor noticed unmistakable symptoms of meningitic disease. Dr. Bridge now saw the child, and a day or two following I was requested to visit her. It seemed to me that there was no question about the diagnosis, and subsequent events demonstrated the correctness of the conclusion. The child die twelve days after the doctor was called to see it.

A *post mortem* was held the following day, at which were present Drs. Danforth, Duncan and the writer. The body was not very much emaciated; the disease having run an unusually rapid course, the wasting of the body was somewhat less than usual.

*The Head.*—Very slight adhesions were found between the dura mater and the calvarium. The dura mater was opaque and slightly rough, and its usual glistening appearance was gone. Along the line of the sagittal suture, numerous white elevations as large as a bird-shot were seen. The vessels of the dura mater were engorged with blood. Well-marked fluctuation was felt when pressure was made upon the surface of the dura mater. Upon removing the dura mater, the vessels of the arachnoid were found to be greatly distended with blood, and inflammatory products were plainly visible on both margins of the longitudinal fissure. Upon examining the smaller vessels of the arachnoid with a hand-glass, a great number of minute, bead-like nodules were seen around and along their margins. The microscopic structure of these nodules will be described presently. The common vascular plexus of the pia mater was the seat of very extensive tubercular deposit; upon nearly all the smaller vessels masses of tubercle were deposited; some of these masses were too small to be seen with the naked eye; some were so large as

Tubercular Meningitis.

J. [illegible] Allen, Sc.

to present distinctly projecting, nodular eminences upon the periphery of the vessel, but the great majority consisted of deposits from the size of a small pin's head to the size of a medium bird-shot. They generally surrounded the vessel, and were either fusiform or spherical. Each nodule was quite isolated, and seemed to be the product of a special center of growth. The microscopic appearances will be described hereafter. The floor of the lateral ventricle was not particularly changed, but the superior surface of the left posterior cornu was softened. The choroid plexus was very pale and anæmic. The floor of the fourth ventricle was thickened, and the seat of considerable tubercular deposit. The substance of the cerebrum and the cerebellum was slightly softened, but not otherwise changed. The thoracic and abdominal organs were healthy.

I come now to consider the cause, or, if more than one exist, the causes, of this terrible disease; a disease, concerning which Vogel and many others say, that in their experience not a single child has recovered—that the prognosis is absolutely fatal.

1st. Undoubtedly the great majority of these cases arise from one cause, namely, the presence of tubercular deposit in some other part of the body. A local cheesy deposit is usually found somewhere, aud tubercular meningitis is part of a general tubercular disease. From the local point of infection, which may be in the lungs or bronchial glands, or any enlarged lymphatic, there is a distribution, either by way of the blood vessels or lymphatics. In the disease under consideration, it is quite probable that the blood is the means of conveyance, for, as we shall see, the lesions are largely in the vicinity and involving the smaller vessels of the base of the brain.

There can be no doubt but that a great majority of deaths from tubercular meningitis take place in families infected from hereditary taint. Sometimes an entire family is lost, the younger members from this disease, and the elder from tubercular troubles as we usually see it developed in adults. Under strict hygienic surroundings the little ones who have received the taint from parents may remain for a long time without its development—indeed it may never be developed, or, as I believe, it may be removed.

2d. While it may be thought that I am retrograding instead of advancing, it certainly seems to me that we are each year accumulating evidence which will ultimately make it certain that tubercle may be propagated by infection. That those intimately associated with the tuberculous often die of the disease, we cannot doubt, and there are now excellent grounds for the opinion long since expressed by Waldenburg, that minute particles exhaled and expectorated from the lungs, may be the medium of infection. (Smith.)

3d. A certain number of children whom I have seen die from tubercular meningitis, have presented no signs of the tubercular or scrofulous diathesis. It is true that I have not had the opportunity of making a post mortem in all these cases, but I have presented the results of one, which, although not enough to form any conclusion whatever, yet in connection with what others have said and observed shows, at least to me, the possibility of this disease developing in those who have no taint whatever, and who have never been exposed to those causes which may propagate the disease by direct infection.

Some one has said that tubercle is simply nodified *connective tissue*, and the experimental investigations commenced by Villemin in 1865, and followed out by Burdon Sanderson, Wilson Fox, Cohnheim and others, seem to demonstrate the fact that artificial irritation beneath the skin, producing an inflammatory product, may, after the lapse of time, by disseminating inflammatory lesions, produce in various organs and tissues, products which present a special tendency to become caseous. Not only is it found that the introduction of certain pathological products, such as tubercle, thickened pus, etc., will produce this result, but finely divided foreign substances not animal, as aniline blue, and traumatic irritations under the skin giving rise to inflammation, such as the use of the seton, will and have produced tubercle in the lung or other organs.

If these discoveries stand the test of future investigation, and are demonstrated to be true, they will be of great importance to us in tracing the causes of different forms of tuberculosis which have hitherto been obscure. The procedure in an experimental case is something like this. A minute particle of this inserted substance is carried to the lung or brain. At the point of deposit an inflammation, and abnormal rapidity of cell proliferation takes place, a product of rapid and imperfect growth is formed, atypical cell development and the so-called tubercle is the result. A child falls from its bed, or down a few stairs, or from a trunk or box, and strikes its head. It may vomit and give some slight evidence of cerebral disturbance, but it is soon forgotten. A very slight injury, not noticeable, was produced, a few drops of pus, or a small coagulum formed, or perhaps only nutrition to a certain part of the brain delayed or disturbed for a short time. It was the *trauma*, however, from which a slight deposit was carried, or around which rapid cell proliferation took place, the formative irritation has occurred, connective tissue has become modified and tubercle may be the result.

That a slightly traumatic injury in a perfectly healthy individual, without the peculiar susceptibility of which we hear so much, will produce tubercle we cannot of course say with certainty. It has seemed to have taken place in a few of my patients. But, with the least scrofulous taint in a child's system, if passed unheeded and no attempt is made to avert its development, no one thing is more clear than that a multitude of diseases, such as whooping cough, pneumonia, catarrh following measles, etc., are capable of rendering certain tissues cheesy, and places from which absorption may take place; and traumatic lesions of the extremities, joints or periosteum, with this tendency, are all that is necessary to produce the abnormal transformation of which I have spoken.

But not to extend to any further length the possible way by which tubercle can be formed in the brain, I will conclude what I have to say in regard to the pathology of tubercular meningitis by quoting from Dr. Danforth's paper, already referred to, the conclusions suggested by his own and other observations:

"1. The lesions peculiar to tubercular meningitis are necessarily, if not exclusively, confined to the smaller blood vessels of the membranes (especially the pia mater) and of the brain substance; and all other lesions are probably secondary to those above mentioned, and are directly or indirectly produced by them.

2. The lesions in question are *always* exterior to the tunica interna, and therefore have no direct structural relation to the circulating blood.

3. The primary seat of pathological activity in tubercular meningitis, is in the perivascular spaces or canals, which surround the smaller blood vessels of the brain and its membranes, as described by His and others; and in its early stages at least, is essentially inflammatory in its nature, the inflammation being at first limited to the meninges.

4. As a consequence of the initiative hyperplasia, which results from, or rather forms a part of the process of the inflammation, an abnormal rapidity of cell proliferation takes place within the peri-vascular spaces; in other words, the leucocytes or bioplasts (Beale) contained in the lymphatic channels, are produced with unwonted haste, and therefore with corresponding imperfection, and the product of this rapid and imperfect growth is the so-called tubercle."

### *Diagnosis.*

It does not appear to me that we have any one symptom pathognomonic of this disease, and it will be only by taking into consideration the entire group of symptoms with the hereditary predisposition, or the history of some traumatic irritation, that we can come to a safe diagnosis. With the cerebral diseases with which tubercular meningitis may be confounded, I cannot stop to speak; nor can I take your time in speaking of any resemblance tubercular meningitis in adults may have to typhoid fever. Typhoid is such a remarkably rare disease among young children in our country, that our diagnostic skill in this respect will hardly be taxed to any extent.

Remittent fever is the disease, more than all others in this climate which will be found to simulate the first stage of tubercular meningitis. The periodical fever, the vomiting and the indifference, almost drowsiness, which we notice in this fever, is exceedingly difficult to distinguish from the stage of tubercular meningitis I have mentioned. We usually find more restlessness and the peculiar cerebral vomiting, and some symptoms referable to the eyes in the meningitic disease. Undue vomiting for several days, and the persistence of the periodical fever after treatment with quinine, give me uneasiness, and in these cases I am always fearful that I am to have developed a more severe disease than any of those of malarial origin. Cases will frequently occur in which our diagnosis will have to be suspended for a few days.

The differential diagnosis between simple and tubercular meningitis, although exceedingly difficult and sometimes absolutely impossible, may be best understood by the following synoptical table:

| *Tubercular Meningitis.* | *Simple Meningitis.* |
|---|---|
| 1. For several weeks the child has emaciated. It has lost its strength and it has become peevish and irritable. | 1. No prodromal period. |
| 2. The child has in the majority of cases either scrofulous or tubercular antecedents. | 2. Healthy progenitors. |
| 3. May have cheesy degeneration from previous disease, or necrosis. | 3. No previous disease. |
| 4. Invasion. Symptoms obscure; periodical fever; vomiting; generally no local symptom for several days. | 4. Invasion. Symptoms *acute;* intense continued fever; vomiting; violent headache, with great heat. |
| 5. No convulsions in many cases until second stage; respiration unchanged. | 5. Convulsions usually from the first; respiration very hurried. |
| 6. The disease has every appearance of being mild and easily amenable to treatment. | 6. The disease has appeared severe and dangerous from the outset. |
| 7. Course from this time generally slow. The fever becomes continued; pupils changeable. Symptoms of brain pressure usually appear slowly; pulse irregular; coma; paralysis. | 7. Course rapid; convulsions may take place with quick succession, and death result in from one to three days from the commencement of the attack. |

The *tache cérébrale* is present in both diseases, and nothing pathognomic can be determined from it.

The ophthalmoscope has been used as an instrument of diagnosis in these diseases, but except as confirmatory of the general symptoms, it is not regarded of great value. In acute disease: "Stasis in the retina alone, indicates only general swelling of the whole brain; positive neuro-retinitis points to an inflammation at the base" (Huguenin).

Congestion and œdema of the optic papilla and surrounding tissue, with tortuosities of the retinal vessels, are more frequent in tubercular than in simple meningitis, since the inflammation and exudation more frequently involve the base of the brain; but diagnostic points of value in this disease can only rarely be made, as the symptoms observed are so nearly allied to those seen where tumor or abscess of the brain exist.

*Treatment.*

From what has already been said, it is inferred that the majority of observers believe this to be almost, if not entirely, a uniformly fatal disease. Of course I cannot here discuss the possibility of certain cases which have been diagnosticated as tubercular meningitis, and have recovered, being simple meningitis, or simple basilar meningitis. We know to a certainty that in almost every case in which the disease is fully developed, death is the final result, and that only in prophylaxis can a physician obtain any results at all satisfactory. The presence of the tubercular or scrofulous diathesis may in a great number of cases be removed, if one could but have the hearty co-operation of the parents. Mothers with this peculiarity should be informed in regard to the probable taint they are to communicate to their offspring, and everything in the way of nourishment and nutritious medicaments should be administered. It is quite possible that such mothers should not be allowed to nurse their children, although I am always unwilling to deprive any child under the year of its proper maternal food—"a food which nature does not afford, nor can art supply a substitute"—unless there is some grave reason for such deprivation. Children in whom there is the faintest possibility of such a taint, should be given the benefit of the country air, if possible—good hygienic surroundings, perfect nutrition—especially a sufficient dietary, for, must I say it, in this blessed land of ours, children do starve to death. Diseases which produce a tendency to the diathesis I have spoken of, should be prevented by avoiding the opportunities of receiving the contagion into the system. I know it is wholly impossible to prevent all children from coming in contact with whooping cough, measles, diphtheria and the like; but there is no reason why the effort should not be made with this peculiar class of children.

They should have the benefit of cod liver oil and syrup of the iodide of iron — the malt and hypophosphate preparations. Above all we should not, because a child is slightly tubercular or scrofulous, despair of either staying the disease or of ultimately restoring to the anxious parents a healthy and robust child. The hopeless pathology of fifty years ago, indeed, gave no hope, but more recent conclusions give a brighter future. While it is doubtful if tubercle in the brain is ever cured, it has been abundantly shown by Niemeyer and others that in other parts of the body it is amenable to treatment, and that by several modes recovery does take place. Rokitansky gives us three methods by which active tubercles may disappear, and there are many reasons why we should attempt the removal, or at least render inert these primary deposits, and thus remove from our patients the danger of secondary deposit and tubercular trouble within the brain. We should not wait till we believe tubercle is deposited, but from the moment we are satisfied nutrition is impaired, or in a condition where tubercular deposit may take place, we should commence treatment. I cannot take the time of the society by enumerating the particular combinations of medicine useful in these cases, or a categorical display of foods suitable in quantity and quality for these little patients. Indeed it would be quite gratuitous to enter into details in the presence of gentlemen so many years my senior. One suggestion and I conclude. It is generally believed that children are not injured by falls and contusions which in older persons would be quite noticeable. Undoubtedly in the main this is true, but if the recent experiments in traumatic irritation are established, it will be well for us as physicians — indeed, our duty — to inform our patrons that it is not absolutely necessary, in order to have a superior intellect developed in a child, that it be encouraged to fall from its bed, or from the tree in the back yard, or allowed to roll down the stairs as a pastime. Traumatic injuries should be avoided — not that one in a hundred die from them, but to avoid the death of this one, which may be the one above all others in which we are interested. It is a high calling to cure disease, to ameliorate suffering; but preëminently more sublime is it to prevent disease, to so change and fortify the youthful constitution which has come into the world maimed and crippled, that its future may be one of health and usefulness.

## Figures and Tables

**Figure f1:**